# An Integrated Model for Circular Waste Management Using the Internet of Things, Semantic Web, and Gamification (Circonomy): Case Study in Indonesia

**DOI:** 10.2196/66781

**Published:** 2025-05-06

**Authors:** Vitri Tundjungsari, Bambang Purnomosidi Dwi Putranto, Muhamad Bahrul Ulum, Nizirwan Anwar

**Affiliations:** 1 Department of Master in Computer Science, Faculty of Computer Science, Universitas Esa Unggul, Jl Arjuna Utara 9, Kebon Jeruk, Jakarta, 11510, Indonesia, 62 8192010576; 2 Department of Information Technology, Universitas Teknologi Digital Indonesia, Yogyakarta, Indonesia; 3 Department of Informatics, Faculty of Computer Science, Universitas Esa Unggul, Jakarta, Indonesia

**Keywords:** IoT, gamification, semantic web, waste management, circular economy, smart bin, Circonomy, semantic, game, gamified, Indonesia, reuse, reduce, recycle, 3Rs, prototype, circular waste, Internet of Things, DSRM

## Abstract

**Background:**

The problem of how to deal with waste is a global issue all countries face. Like many developing countries, Indonesia has inadequate infrastructure to process the extremely high volume of waste produced throughout the country and minimal public participation in proper waste management. Although the Indonesian government regulates waste banks as a community-based waste management solution, there is a lack of integrated technological innovations to support waste banks. This study fills the gap by developing Circonomy, a model combining Internet of Things, gamification, and semantic web technologies to advance community-based circular waste management.

**Objective:**

The aim of this study is to develop Circonomy as a circular waste model that integrates an Internet of Things–based smart bin, semantic web, and gamification as an innovative technological solution.

**Methods:**

We identified the problem by observing Indonesian waste banks at 3 locations in Jakarta and Yogyakarta to define and design Circonomy. The Circonomy prototype was developed using the Design Science Research Methodology and evaluated based on technical performance and user experience. The technical performance has three indicators: bin capacity accuracy with a minimum of 80% precision, bin lid response time <5 seconds for a minimum of 80% of trials, and data transmission success rate of at least 80%. The user experience metric has two indicators: a minimum of 80% reporting high usability and ease of use, and at least 80% of users reporting that they feel more motivated using the prototype than the traditional waste bank. We selected 10 random participants aged 18-60 years to perform a user experience evaluation of our prototype.

**Results:**

The Circonomy prototype demonstrated sound and stable performances related to technical performance and user experience. Circonomy achieved at least 80% technical performance accuracy, comparable to industry standards. The accuracy problem lies in the placement of the ultrasonic sensor. The waste should be placed directly under the ultrasonic sensor to ensure the bin’s capacity measurement accuracy. The user experience testing results from 10 participants indicated that Circonomy has excellent user engagement, and 100% felt motivated by gamification and 80% found the mobile app easy to use.

**Conclusions:**

The testing results showed that Circonomy has acceptable performances for early-stage prototyping, with at least an 80% accuracy rate in technical performance and user experience. This ensures that Circonomy operates effectively in real-world conditions while remaining cost-efficient and scalable. For future development, Circonomy will prioritize enhancing the accuracy and reliability of sensor-based occupancy detection through improved sensor placement, the integration of multiple sensors, and an exploration of alternative technologies for regions with limited IT resources. In addition, more gamification features such as challenges and quizzes should be added to improve user experience and motivation.

## Introduction

### Background

The waste problem is complex and presents significant environmental, social, and economic challenges worldwide. It is characterized by increasing waste production, inadequate waste management systems, and the ecological impacts of waste disposal. Rapid urbanization, population growth, and industrialization, especially in developing countries, are mitigating this problem. Some of the root causes of the global waste problem are capitalist consumption patterns [1], ineffective waste management systems [2,3], and hazardous waste disposal [4,5]. Developing countries such as Indonesia face persistent challenges, including limited infrastructure, inefficient waste handling processes, low community participation, and inadequate awareness of proper waste sorting and disposal processes [2,3,5]. In contrast, developed countries, such as those in Europe [6] (including Germany [7] and Spain [8]), have successfully embedded responsible waste management practices in the culture of their communities through effective incentives.

Based on data collected from 341 districts and cities throughout Indonesia in 2023 released by the National Information System for Waste Management (Sistem Informasi Pengelolaan Sampah Nasional) and the Minister of Environment and Forestry [9], Indonesia generates 37,811,293.71 tons of waste annually, of which only 62.54% can be managed and 13.64% can be reduced per year. The data show that Indonesia urgently needs strategies and innovations to improve waste reduction, handling, and management.

We identified several potential solutions and opportunities to address the waste problem:

1. Integrated waste management systems: Integrated waste management systems, comprising reduce, reuse, recycle (3R) strategies, can significantly mitigate waste-related issues. These systems require coordinated policies, financial support, and infrastructure development [3,10].
2. Innovative waste disposal technologies: Most existing studies typically investigate the use of Internet of Things (IoT)–based smart bins [11-15], integration of IoT and artificial intelligence algorithms [16], gamification to encourage recycling behaviors [17,18], and semantic web technologies for enhanced data integration [19]. Microbial waste utilization and mobile waste processing plants are also used to enhance waste management efficiency and reduce the environmental impact [20].
3. Community-based initiatives: In Indonesia, community-based initiatives have been started and promoted by the government with the Regulation by the Ministry of Environment and Forestry Number 14 of 2021 concerning Waste Management at Waste Banks [21], which emphasizes community-driven, integrated, and circular economy–focused waste management solutions. Nikmah et al [20] discussed the success story of waste banks, which uses an eco-enzyme and demonstrates the potential of community-based solutions to transform organic waste into valuable products and promote environmental sustainability. Eka et al [22] discussed community involvement in waste management through waste banks, which are run by the community to enhance waste segregation, handling, and management. Effendi et al [23] developed a desktop-based waste bank information system as a tool to support waste bank activities in Indonesia.

From a review of the literature, we found that there are some research gaps:

1. Lack of integrated technological solutions: Some innovations that are often found in the literature to support waste management are IoT-based smart bins, gamification, and semantic web. These innovations have generally been applied separately rather than integrated into a comprehensive circular waste management model. Our proposed model addresses this gap by integrating these 3 elements into a cohesive system designed specifically for community-based waste management operations.
2. Limited technology adoption in community-based circular waste systems: Previous waste management solutions often focused on municipal- or city-level waste management and neglected community-managed operations such as waste banks, which are particularly prevalent in Indonesia. Most studies rarely address how technology can support local waste banks as a community initiative along with automated processes, incentivization, and participation. There are some studies related to technological support waste bank management systems within Indonesia. However, none of them propose a comprehensive approach combining automated waste bank innovations and increasing community motivation to take action in circular waste management. Our proposed model deals not only with how technology and innovation can be used for circular waste management but also with how to engage the community and deliver social impact to all stakeholders. We address the gap by using technology and the concepts of IoT, gamification, and semantic web to provide real-time semantic interconnections between stakeholders and waste data and enhance data-driven decision-making at the community level.
3. Limited use of gamification in circular waste management systems, especially in Indonesia: Indonesia has a regulation about waste banks (Number 14 of 2021 concerning Waste Management at Waste Banks [21]), emphasizing community-driven, integrated, and circular economy–focused waste management solutions. Our proposed model explicitly targets this gap by automating local operational processes such as waste weighing and recording integrated with points, leaderboards, and rewards schema using a gamification approach and technology-driven automatic process, with real-time feedback, explicitly tailored for community-based settings. We also address the gap by encouraging recycling behaviors and integrating it with the circular economy.

In short, Circonomy addresses these critical gaps by integrating IoT, semantic web, and structured gamification into a unified system tailored for community-managed waste management. This significantly improves operational efficiency and enhances stakeholder participation within the circular economy framework.

### Objectives

This research aims to address the waste problem in Indonesia by introducing Circonomy, an innovative circular waste management model. This research makes the following primary contributions to the literature and practice of circular waste management:

1. Integration of technologies: It combines IoT-based smart bins, semantic web technology, and gamification into a unified, community-centered system, uniquely enhancing operational efficiency and community engagement simultaneously.
2. Automation of waste bank processes: It aligns with the applicable Indonesian regulation, addressing limitations of manual processes (eg, inaccurate weighing, data entry errors). Real-time IoT sensor data and semantic web integration optimize waste management and decision-making.
3. Structured gamification approach: This approach implements incentive-based gamification (points, leaderboards, rewards) to motivate sustainable 3R behaviors and foster continuous community participation.

The specific objectives targeted by this research are as follows:

1. Integrated technological solution: develop a comprehensive, technology-driven circular waste management model that automates traditional waste bank operations (addressing gaps 1 and 3).
2. Real-time data collection and accuracy: develop an IoT technology that facilitates accurate real-time data collection (addressing gaps 1 and 2).
3. Semantic web integration: enhance operational and decision-making efficiency by enabling real-time stakeholder interactions and optimizing waste treatment processes (addressing gaps 1 and 2).
4. Enhanced community engagement through gamification: encourage sustainable behaviors through structured gamification incentives explicitly tailored for local communities (addressing gaps 1 and 3).
5. Scalability and adaptability: provide a flexible model that can be deployed across various socioeconomic contexts, including resource-constrained environments (addressing gaps 1 and 2).

## Methods

### Overview

**Figure 1.**
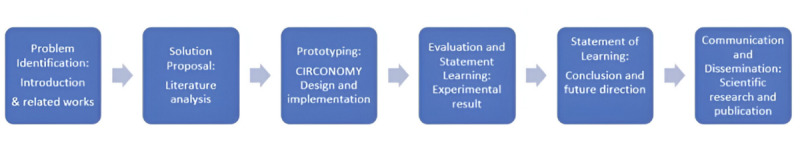
The stages and activities of the DSRM (adopted from [24]). DSRM consists of 6 main stages: problem identification, solution proposal, prototyping, evaluation, statement of learning, and communication and dissemination. DSRM: Design Science Research Methodology.

The Design Science Research Methodology (DSRM) is used in this research. It consists of six main activities [24]: (1) problem identification, (2) solution proposal, (3) prototyping, (4) evaluation, (5) statement of learning, and (6) communication. Figure 1 shows the DSRM stages and activities.

Our decision to use DSRM aligns with our objectives involving integrated waste management, innovative circular waste technology, and community-driven initiatives. DSRM has been used widely in several smart city innovation projects. For example, Effendi et al [23] developed a desktop-based waste bank information system in Indonesia. Henaien et al [24] applied DSRM to develop smart waste management systems, further validating its efficacy in creating impactful solutions for environmental management. Similarly, Lapão et al [25] implemented the Smart Public Health City framework in Europe using DSRM. Duque [26] successfully used DSRM for IoT-based smart city innovations, demonstrating how the methodology effectively addresses urban technological challenges. Moreover, DSRM explicitly supports iterative development, rigorous evaluation, and clear dissemination. Duque [26] mentioned in his research that DSRM is a methodology that allows for the production of innovative prototypes and can be used to solve an organization’s problems.

### Problem Identification

At this stage, our research project investigated the waste problem faced by Indonesia and how current waste management–related regulations propose to solve it. The Indonesian government regulation as agreed in the Regulation of the Ministry of Environment and Forestry Number 14 of 2021 concerning Waste Management at Waste Banks [21] states that: “(a) Waste management should be comprehensive and integrated, applying circular economy principles collaboratively by government and society to achieve economic, health, and environmental benefits.”

“Waste management is as intended in point (a) can be done synergistically through the waste bank.” A waste bank is defined as a facility for managing waste with the 3R principle, as well as educational facilities, behavioral changes in waste management, and the implementation of a circular economy, which is formed and managed by communities, business entities, and local governments.

To identify the problem, we observed 3 waste banks located in 2 different cities in Indonesia (Jakarta and Yogyakarta).

### Solution Proposal

**Figure 2.**
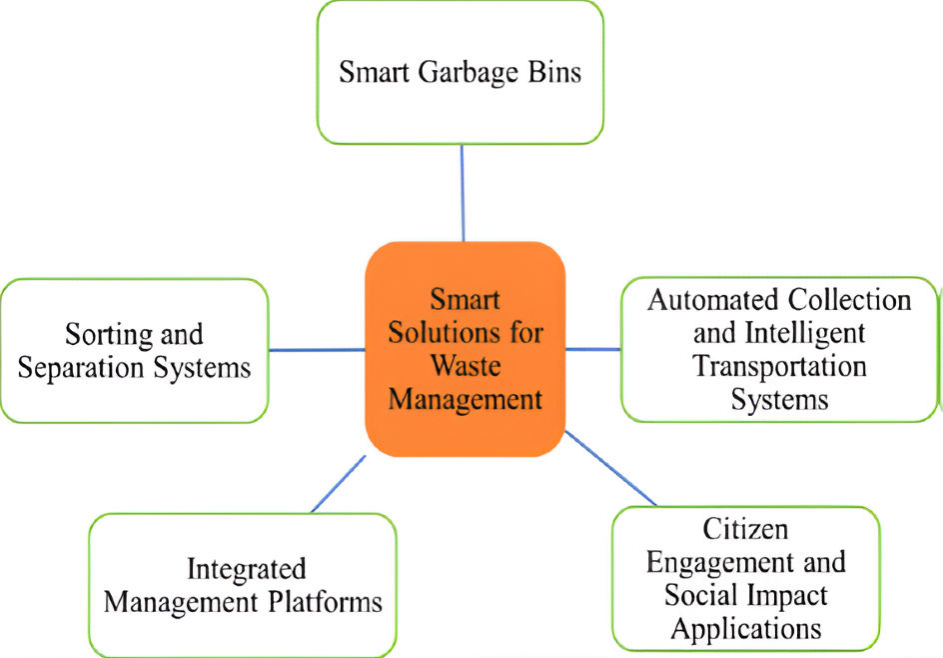
Classification of smart city solid waste management systems [24]. There are 5 classifications: smart garbage bins; sorting and separation systems; automated collection and intelligent transportation systems; integrated management platforms; and citizen engagement and social impact applications.

Henaien et al [24] mentioned that there are five smart city solid waste management system classifications: (1) smart garbage bins, (2) sorting and separation systems, (3) integrated management platforms, (4) automated collection and intelligent transportation systems, and (5) citizen engagement and social impact applications. This research is a pilot project aiming to develop a first-stage prototype based on the Circonomy model. In developing the first stage of our prototype, we only focused on the design and development of (1) smart garbage bins, (2) citizen engagement mechanisms, and (3) integrated management platforms regarding circular waste management. This is in accordance with the objectives we want to achieve, as we discussed in the Introduction. This targeted focus was chosen due to the particular relevance and effectiveness of these 3 areas in addressing the identified waste management challenges in Indonesia and related countries. Furthermore, those 3 focuses align with Indonesia’s regulatory framework, particularly the Regulation of the Minister of Environment and Forestry Number 14 of 2021 concerning Waste Management at Waste Banks, which emphasizes community-driven, integrated, and circular economy–focused waste management solutions. In the development of the second-stage prototype, the features of (1) sorting and separation functions and (2) automated collection and intelligent transportation systems will be added, resulting in a comprehensive integrated circular waste management system. Figure 2 shows the smart city solid waste management classifications [24].

### Prototyping

This phase is crucial as it involves creating prototypes that demonstrate the practical application of our proposed hardware and software solutions. Our prototype, a smart bin, serves as an automated waste collection and weighing tool in our model. We tested the prototype-based Circonomy model using a combination of technical tests, user experience feedback, and preliminary impact projections. The primary objective was to assess the model’s automation capabilities, data transmission reliability, and user engagement via gamification incentives. Controlled experiments were conducted in multiple scenarios, focusing on (1) automated bin operations (lid opening/closing based on user interaction); (2) automated weighing operations (ensuring precise tracking of deposited waste); (3) bin capacity estimation (evaluating ultrasonic sensor effectiveness); (4) data transmission and processing (assessing server communication and response times); and (5) gamification effectiveness (measuring user engagement based on points, rewards, and leaderboard interactions).

### Evaluation and Statement

To carry out the testing process, we needed to determine the workflow scenario of our proposed model. The workflow is described as follows:

1. Installation of Circonomy app: The user downloads the Circonomy mobile app.
2. User registration and authentication: Users register and log in via the Android app. User data are stored on the server.
3. Connecting to smart bin: The user selects the bin by scanning the QR code and then sends a command to open the lid.
4. Bin lid control: Based on commands from the app, the ESP32 system-on-a-chip microcontroller controls the servo motor to open or close the bin lid. The lid will close automatically after the waste is thrown into it.
5. Collecting waste data: After the bin is loaded, the load cell and ultrasonic sensor measure the weight and capacity of the bin, and the data are sent to the ESP32. The bin’s capacity data are calculated by measuring the distance from the bottom of the bin to the lid, and then converting it to a percentage using an ultrasonic sensor. For example, if there is plastic bottle waste with a height of 10 centimeters thrown away in a bin with a depth of 20 centimeters, then the bin capacity is 50%. The LED will show the weight and bin capacity after the measurement.
6. Sending data to server: The ESP32 sends waste weight and bin capacity data to the server via the REST application programming interface (API). The waste’s weight is measured using a load cell sensor, while the bin’s capacity is calculated using an ultrasonic sensor. The server processes the data received, converts waste weight into points, and updates the leaderboard and history data.
7. Feedback to user: The mobile app displays the points earned on the user profile and the leaderboard and updates the bin’s status on the web-based application.

To measure the testing results, we used the following evaluation metrics:

1. Technical performance indicators consisted of three metrics: (1) bin capacity accuracy (minimum 80% precision); (2) bin lid response time (<5 seconds in at least 80% of trials); and (3) data transmission success rate (minimum 80%).
2. User experience metrics were gathered from 10 users, with two targets: (1) minimum 80% reporting high usability and ease of use and (2) at least 80% of users feeling more motivated using the prototype compared to the traditional waste bank. We selected 10 random users aged 18 to 60 years to be included in prototype user testing. Cazanas et al [27] mentioned in their research that 10 users are enough to achieve 90% problem discovery on an app’s usability.

### Ethical Considerations

This research involved the development and evaluation of the Circonomy model, which was designed for community-driven circular economy initiatives. Ethical considerations were diligently addressed throughout the study. The study protocol underwent an ethical review process by the Esa Unggul University Ethical Board, which determined that the research qualified for an exemption due to minimal risk and the use of anonymized secondary data. Participant privacy and confidentiality were strictly maintained through anonymization protocols. No personal identifiable information was collected or disclosed and no compensation was provided to participants.

## Results

### Problem Identification

We investigated how waste banks run by doing a case study in some provinces in Indonesia (Daerah Khusus Jakarta and Daerah Istimewa Yogyakarta). Here are some findings obtained from our observation of three waste banks:

1. Waste banks have common workflows, which is in line with previous research [28]. The workflows of the waste banks in Indonesia consist of several activities: (1) waste separation and segregation, (2) waste deposit, (3) waste weighing, (4) waste recording and ledgers, and (5) waste collection for further treatment.
2. There are some common problems faced by waste banks, such as (1) inefficiency of waste bank management, especially when handling the waste due to uncertain working hours; (2) lost and incorrect waste recording documents; (3) lack of staff at the waste bank; (4) slow processes in waste weighing and no automated record (manually handwritten) in the waste book ledger; (5) people are often reluctant to come to the waste bank because the process and surrounding environment are not hygienic; and (6) people are not interested in supporting the waste bank because there are minimal incentives and rewards.

### Solution Proposal: Circonomy

**Figure 3.**
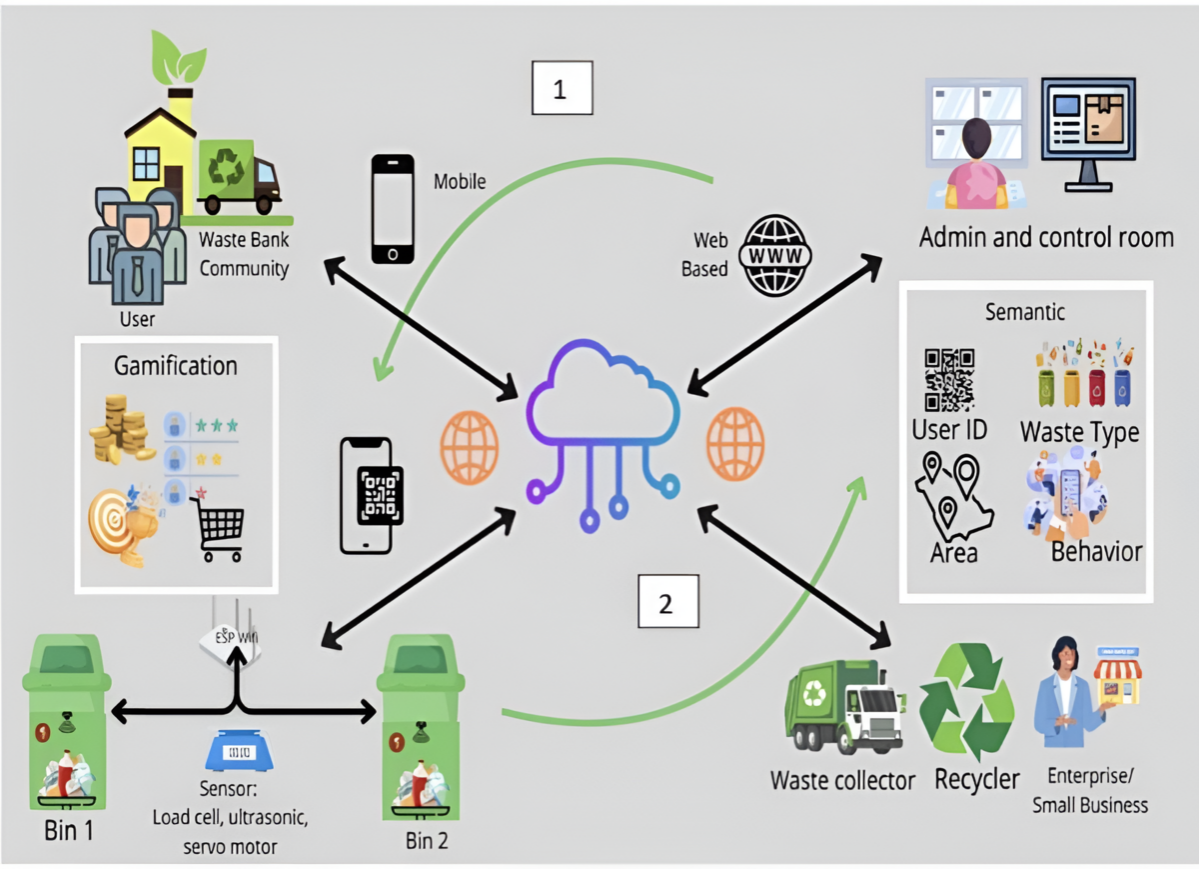
The proposed Circonomy model. Circonomy has two main workflows: (1) waste treatment and disposal, in which the user (individual or waste bank community) puts the garbage in the smart bins using the mobile app, which is enhanced by gamification features; and (2) garbage collection by a waste collector, recycler, enterprise, or small business and monitoring by an administrator. This workflow is supported by the semantic web concept to enhance reduce, reuse, and recycle behavior.

Our proposed model, Circonomy, is inspired by the circular economy concept. The circular economy contains different elements required for the transformation from a linear to a circular system, which includes: (1) design for sustainability, (2) cleaner production, (3) reverse logistics, (4) industrial ecology, (5) energy efficiency, (6) waste management, (7) product life extension, and (8) product as service [29]. Circonomy focuses on the waste management element, which consists of two subelements: (1) waste treatment and disposal and (2) reduce, reuse, and recycle. The model is presented in Figure 3.

As we can see from the model, there are cycles described by the numbers 1 and 2. Workflow 1 is the process involved in the waste and treatment disposal subelement, while workflow 2 is engaged in the 3R subelement. There are three main actors in our proposed model: (1) the community and users of the waste bank (the waste disposers), (2) the system administrator or operator, and (3) the waste collector, recycler, enterprise, or small business. The model has three main components: (1) smart bins, (2) a mobile app, and (3) a web-based application.

Gamification and the semantic web approach are used in our model to enhance user engagement in circular waste management by having these features:

1. User profiles and points: Users are categorized into residents and businesses. Users can create user profiles linked to waste collection and recycling activities.
2. Points and leaderboard: Users receive points for correctly placing waste in the smart bin, reducing waste, and participating in community cleanups. The points gathered by each user are displayed on the leaderboard in rank order.
3. Rewards: Points can be redeemed for rewards, for example, to get discounts at local eco-friendly stores or from other third parties.
4. Cross-platform data sharing: Waste data from municipalities, manufacturers, and recycling centers can be standardized and shared across platforms, providing better visibility into product life cycles. This enables real-time insights into waste flows and statuses to decide on the best disposal or recycling options based on local conditions.

### Prototyping

**Figure 4.**
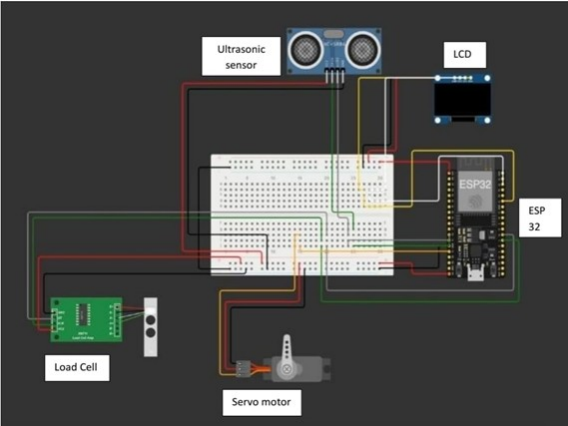
The Internet of Things component in Circonomy. This component consists of the following: (1) an ultrasonic sensor to measure the bin’s capacity; (2) a load cell to measure the waste’s weight; (3) a servo motor to open or close the bin’s lid automatically; (4) an ESP32 to serve as microcontroller, which sends and receives sensor data over a WiFi connection; (5) an LED to display the waste’s weight and bin’s capacity data.

**Figure 5.**
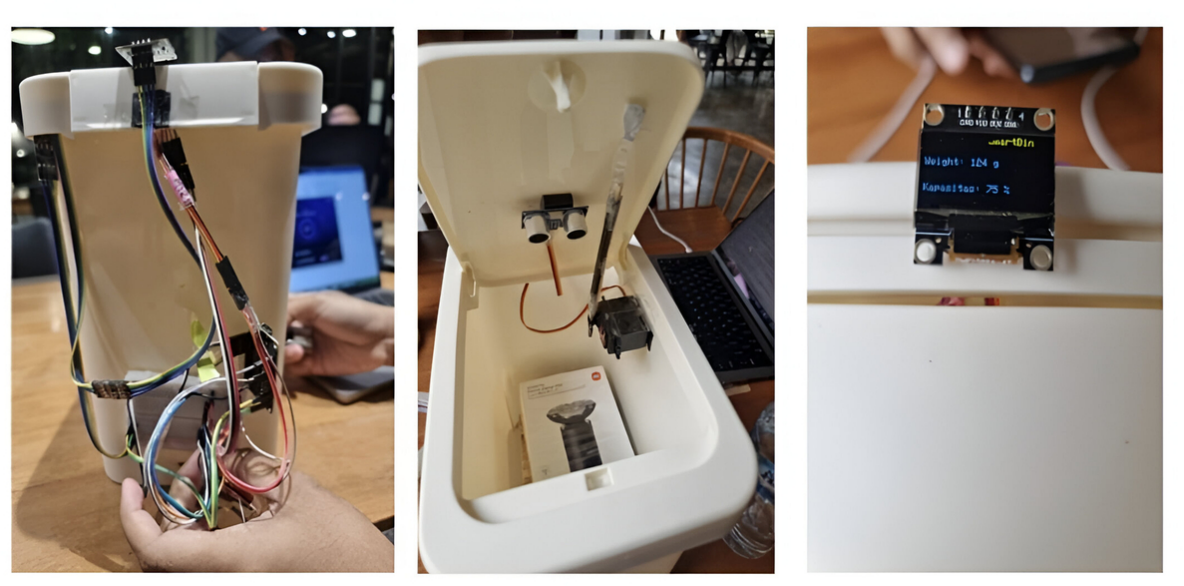
The prototype of the Circonomy smart bin. The prototype connects hardware and software through the node.js/Express REST API server to handle communication between the ESP32 in the smart bin and the Android app. ESP32 serves as a microcontroller to communicate with other modules (such as the load cells, ultrasonic sensors, and servo motors) using the REST API server to send and receive data. API: application programming interface.

To design the smart garbage bin in our model, we separated it into hardware and software components, as follows:

1. Hardware components (IoT). These components form the smart garbage bin by sending waste bin data to the software component. The hardware component consists of the following: (1) ESP32, which acts as the main controller of the IoT system. ESP32 will integrate and control other modules, such as load cells, ultrasonic sensors, and servo motors. Additionally, the ESP32 will communicate with the REST API server to send and receive data. (2) A load cell, which measures the weight of waste placed in the bin. These data are sent to the ESP32 for further processing, as well as to the ultrasonic sensor, servo motor, and LED. (3) The ultrasonic sensor measures the distance between the bin and the sensor to determine the level of bin fullness. These data are converted into a percentage of occupied capacity. (4) A servo motor is used to open and close the bin lid based on commands from the Android app sent via the REST API server to the ESP32. (5) The LED displays the information on the waste bin. Figure 4 shows the IoT components in our prototype.
2. The software components in this model include a server and API, an Android app as an interface to the user, and a web-based application as an interface to the administrator. We used the Node.js/Express REST API server to handle communication between the ESP32 and the Android app. Functions included (1) user management (registration, authentication, and user data management); (2) smart bin management (registration and monitoring of each connected bin); (3) reward and challenge management (manage rewards and challenges in which users can participate); (4) leaderboard management (save and display rankings based on points from collected trash); (5) history data (stores waste weight data from each connected bin); and (6) mobile apps and web-based applications with features, for example, connecting to a smart bin so that the user can select a bin to dispose of trash, waste disposal history so that the user can view the weight of the waste automatically after he/she puts the waste in the bin, monitoring and control so that the application sends commands to the server to open the bin lid and presents the waste’s weight and the bin’s capacity on the LED, points and rewards systems so that users can view their points and redeem rewards, and a leaderboard to display user rankings based on collected waste points. Figure 5 presents the smart bin prototype, which was developed based on the Circonomy model.

**Figure 6.**
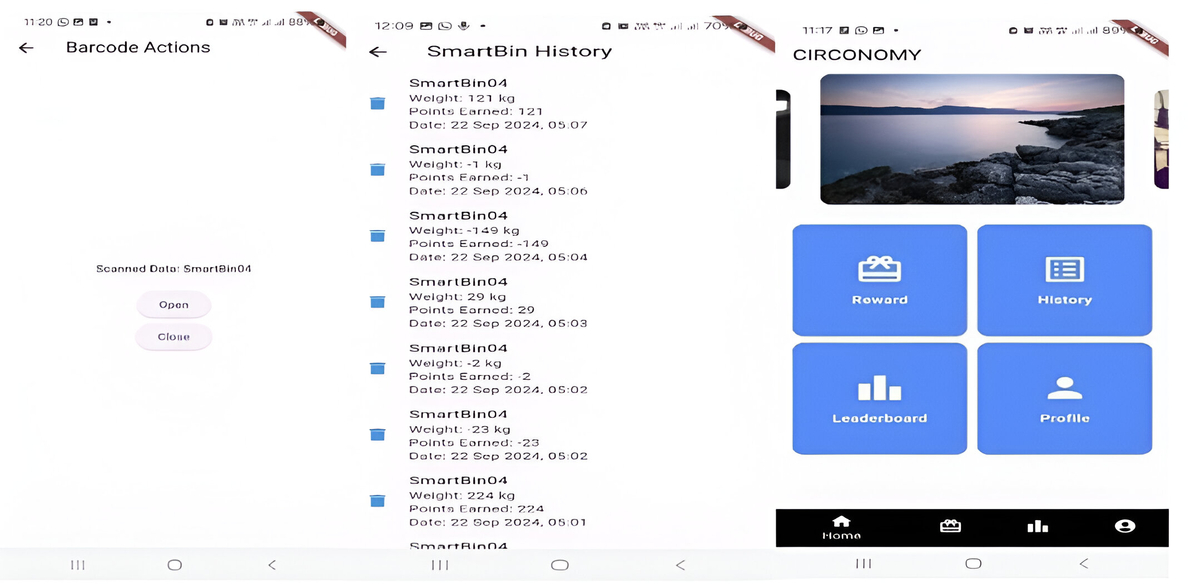
The interface of the Circonomy mobile app. The left figure is the interface of the mobile app used to open and close the bin’s lid. The middle figure is the interface showing the smart bin history (positive values show that waste was placed in the bin and negative values indicate that waste was taken out from the bin). The right figure presents the interface of gamification features (reward and leaderboard) in the mobile app as a reward mechanism to enhance user engagement.

**Figure 7.**
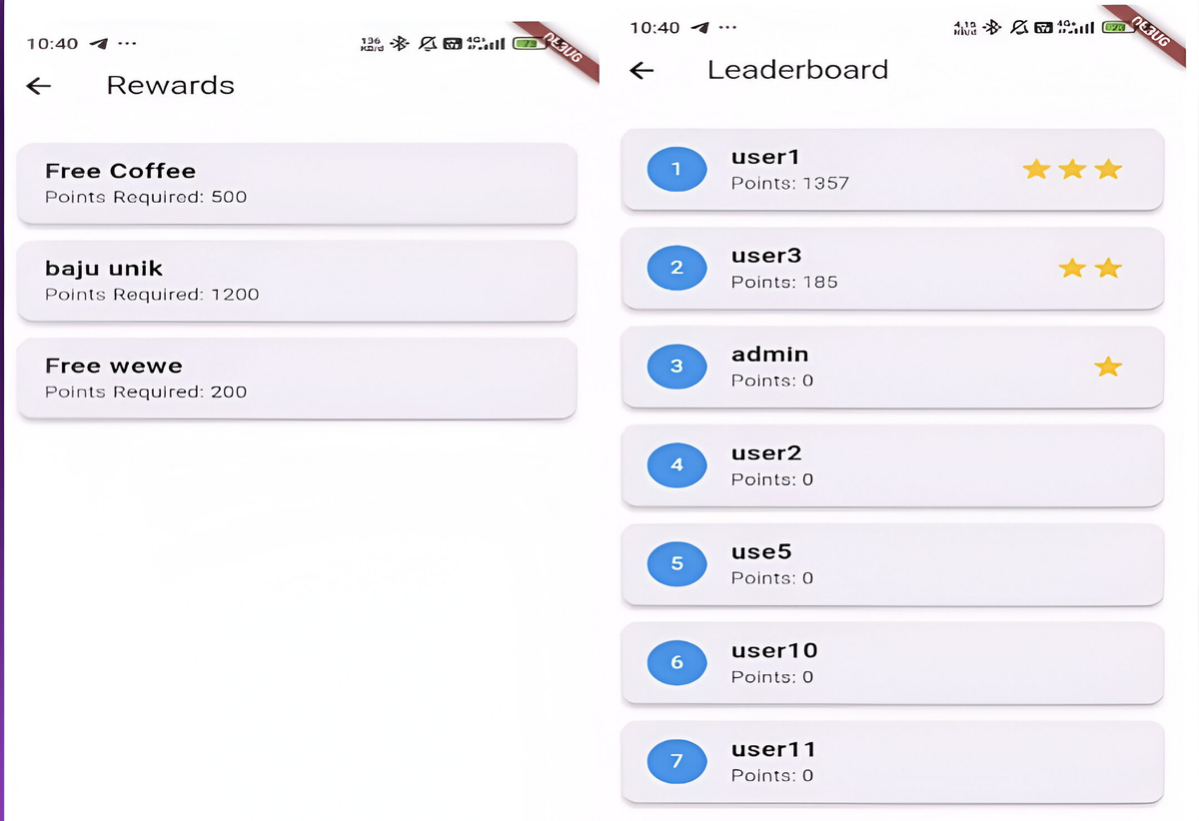
The interface of the Circonomy mobile app: rewards and leaderboard. The interface shows the leaderboard based on the points gained from using Circonomy (right side); these points can be exchanged for rewards such as free coffee, clothes, or others (left side).

The Circonomy model incorporates a gamification system designed to increase user engagement, motivation, and long-term participation in community-based waste management activities. Gamification elements are embedded into mobile apps and web-based applications directly connected to the IoT-enabled smart bin system. The gamification framework aims to encourage sustainable waste behaviors, particularly the 3R practices, through the following mechanisms:

1. Points system: The point system is triggered every time a user deposits waste into the smart bin. The system automatically records the type of waste (eg, plastic, organic, paper) and the weight of the waste is measured via IoT-connected load cell sensors. Based on these inputs, the system calculates points for each user. Different waste types may earn different point values to encourage the sorting of high-priority recyclables. The heavier and more frequent the deposits, the more points a user can collect and accumulate.
2. Rewards: Users can exchange accumulated points for various rewards, such as vouchers (for groceries, services, or local products), eco-friendly goods (such as reusable bags or compost kits), or community recognition (through badges or certificates). Figure 6 shows the interface of the Circonomy mobile app with the points and rewards feature.
3. Leaderboards and challenges: The Circonomy mobile app features a leaderboard to visualize users’ point rank from the highest to the lowest. This fosters healthy competition and public recognition. Periodic challenges and events are also introduced to keep engagement high, such as the top recycler of the month, community recycling drives with bonus points, and group challenges where groups compete to achieve collective waste reduction goals. Figure 7 shows the interface of the Circonomy mobile app with the rewards and leaderboard feature.
4. Incentive schema: The gamification system is designed around a structured incentive schema to maintain ongoing motivation, including immediate feedback via notifications after deposits, accumulated rewards over time, and social incentives and badges as participation status in the community.

### Evaluation and Statement

To measure the testing results, we determined some evaluation metrics: (1) technical performance indicators and (2) user experience metrics. Technical performance indicators had three metrics: (1) weighing accuracy with at least 80% precision, with minor discrepancies due to sensor positioning; (2) bin lid response time <5 seconds in 90% of trials; and (3) data transmission with a minimum 80% success rate, affected by network reliability and processing speed. User experience metrics were gathered from 10 users (aged 18-60 years), with two targets: 80% reported high usability and ease of use and 100% found the gamification incentives motivating for waste disposal, with the leaderboard and reward features receiving positive engagement feedback. We selected 10 random users aged 18 to 60 years to perform usability testing on our prototype. The results of the testing are summarized in Table 1.

**Table 1.** Prototype testing results based on technical performance and user experience.

Category and process		Result	Metrics
**Technical performance indicator**			
	Installation of Circonomy mobile app	All users find that the process is easy	100% installation success (10/10 tests)
	User registration and authentication	All users find that the process is easy	100% registration success (10/10 tests)
	Connecting to smart bin by scanning a QR code using the mobile app	All users find that the process is easy	100% successful connection (10/10 tests)
	Open and close the bin’s lid automatically	The result depends on the position of the servo motor, which sometimes moves unexpectedly (still needs further improvements to the design)	90% response time <5 seconds (9/10 tests)
	Collecting waste data and showing weight and bin capacity data on an LED screen	The result depends on the viewpoint of the ultrasonic sensor toward the waste’s position	Ultrasonic accuracy of 80% (8/10 tests). See Table 2 for more detailed measurements.
	Sending data to the server and updating waste history data that have been collected for each user and converting weight to points	The successful rate of data transmission depends on process number 5 and internet speed	80% data transmission success (8/10)
	Feedback to the user by displaying points earned, leaderboard, and the status of the bin on the mobile app and web application	The successful rate of data transmission depends on process number 6 and internet speed	80% data accuracy updating points and leaderboard success (8/10)
**User experience**			
	Usability and ease of use	Most users strongly agree that the prototype has excellent usability	80% (Likert scale 1‐5). See Table 3 for more detailed testing results.
	Gamification incentives are motivating	All users are completely in agreement that Circonomy enhanced motivation	100% (Likert scale 1‐5). See Table 3 for more detailed testing results.

**Table 2.** Numeric results of the bin’s capacity (sample of 10 trials). Net bin dimensions (15 cm width × 20 cm length × 20 cm depth) with a total volume of 6000 cm^3^ or equal to 6000 mL. Displayed bin capacity on the LED is measured from the distance from the top of the waste to the ultrasonic sensor divided by depth of the bin. The ultrasonic sensor is placed under the bin’s lid.

Trial number	Waste type and size	Displayed bin capacity (the waste position should be observed carefully)	Actual bin capacity (%)	Result
1	1 plastic bottle with a height of 20 cm	0% (the bottle is placed under the ultrasonic sensor)	0% available	Accurate
2	1 box with a height of 10 cm	50% (the box is placed under the ultrasonic sensor)	50% available	Accurate
3	1 box with a height of 5 cm	75% (the box is placed under the ultrasonic sensor)	75% available	Accurate
4	1 plastic bottle with a height of 14 cm	70% (the plastic bottle is in a fallen position)	30% available	Not accurate
5	1 plastic bag contains food waste with a height of 5 cm	75% (the plastic bag is placed under the ultrasonic sensor)	75% available	Accurate
6	9 plastic bottle with a height of 6 cm	70% (the bottles are placed and distributed evenly inside the bin)	70% available	Accurate
7	1 box with a height of 15 cm	25% (the box is placed under the ultrasonic sensor)	25% available	Accurate
8	1 plastic cup with a height of 10 cm	100% (the plastic bottle is placed not directly under the ultrasonic sensor)	50% available	Not accurate
9	1 box with a height of 20 cm	0% (the box is placed under the ultrasonic sensor)	0% available	Accurate
10	1 box with a height of 4 cm	80% (the box is placed under the ultrasonic sensor)	80% available	Accurate

**Table 3.** User experience testing results in terms of (1) usability and ease of use and (2) the gamification incentive scheme. Ten respondents rated their opinion on provided questions using a Likert scale (1 to 5).

Topics	1	2	3	4	5	Means	Interpretation
Overall reaction to the system: ease of use and features			2	6	2	4	80% of users agree that Circonomy is easy to use (mean score 4/5)
Enhance motivation with gamification (points, rewards, leaderboard)					10	5	100% of users agree that gamification enhances motivation to use Circonomy (mean score 5/5)

## Discussion

### Preliminary Findings

The Circonomy model successfully integrates IoT, gamification, and semantic web technologies into a circular waste management system, addressing critical gaps in traditional waste bank operations. Our prototype testing demonstrated at least 80% accuracy in waste weighing, bin capacity estimation, and data transmission, aligning with industry standards. User engagement was notably high, with 100% of participants reporting increased motivation due to gamification features, and 80% finding the mobile app easy to use. The structured incentives, including leaderboards and reward systems, encouraged active participation, distinguishing Circonomy from conventional waste management models. Furthermore, the system’s economic feasibility highlights the potential for public-private partnerships to enhance its sustainability. These findings indicate that Circonomy is an effective and scalable solution for optimizing community-based waste management and promoting circular economy principles.

### Model Evaluation

Our model is proven to perform well, with an 80% accuracy threshold. The 80% accuracy threshold for weighing precision, data transmission, and overall system performance is justifiable and acceptable for the following reasons.

#### Acceptable Performance for Early-Stage Prototyping

The Circonomy model is in its prototype and testing phase. At this stage, achieving 100% accuracy is unrealistic due to system constraints such as sensor limitations, environmental factors, and calibration challenges. Most early-stage IoT-based waste management systems report similar performance levels, with accuracy typically between 70% and 85% during initial testing phases [24,26]. An 80% accuracy rate ensures the system functions reliably under real-world conditions while allowing room for iterative improvements in future versions.

#### Sensor and Environmental Constraints

There are several sensor and environmental constraints that need to be taken into account, including the following:

- Weight measurement: The 80% weighing accuracy accounts for minor errors caused by uneven waste placement, bin tilting, or variations in material density (eg, lightweight plastics vs heavy glass).
- Ultrasonic bin capacity detection: How waste settles inside the bin influences sensor accuracy, which may create slight variations in readings.
- Data transmission: The 80% data success rate mainly depends on internet speed and server response times, which can vary due to network congestion or IoT device latency.

#### Comparison With Industry Standards

Similar IoT-based smart waste bin studies have reported performance accuracy in the 75%‐90% range under varying conditions [25], while for waste sorting and collection models, an accuracy threshold of 80% is typically considered operationally efficient in smart city applications [6].

#### Balancing Accuracy With Cost and Feasibility

Achieving more than 80% accuracy would require higher-end sensors and more expensive technology (eg, industrial-grade load cells, lidar-based bin occupancy detection), which would increase system costs and reduce scalability for community-level waste banks.

Thus, we can conclude that the current 80% accuracy rate ensures that Circonomy operates effectively in real-world conditions while remaining cost-efficient and scalable. However, we still require incremental refinements and continue to enhance accuracy performance over time.

### Challenges and Limitations

#### Scalability and Adaptability

Circonomy is designed with IoT, sensor components, and mobile apps that require sound and stable internet connection access, however, we also provide flexible schemas to enable Circonomy to operate in regions with limited IoT infrastructure and intermittent internet connectivity. These adaptations allow the circular economy to remain operational across diverse socioeconomic contexts, from urban centers with full digital infrastructure to rural areas with limited technological access.

The technology flexibility ensures that the benefits of the circular economy and incentivized waste management can be extended to underresourced communities, fostering inclusive participation regardless of technological limitations. To ensure accessibility in such areas, the model incorporates several adaptive strategies:

1. Offline functionality. The mobile app and web application support offline data entry. Waste transactions can be recorded locally on devices and synchronized with the central system once a stable internet connection is available.
2. QR code validation. In low-connectivity areas, QR codes are used to identify users and link waste deposits to their profiles without needing constant real-time communication with the server.
3. Delayed synchronization. Data collected during offline periods are stored locally and batch-uploaded during periods of connectivity, ensuring no loss of transactional data.
4. In other resource-constrained areas with limited availability of IoT, we can exchange IoT with simpler technology, such as manual weighing systems integrated with mobile/web data logging.

#### Sustainability Strategies

To ensure long-term sustainability across diverse socioeconomic contexts, Circonomy incorporates several adaptive strategies that address financial, social, and operational challenges, making the model resilient and scalable in various environments. Community ownership and empowerment should be encouraged through Circonomy to foster a sense of responsibility, strengthen social cohesion, and promote long-term participation, for example, by transferring ownership and daily operational responsibilities to local waste bank members.

Collaboration between local governments, private companies, and nongovernmental organizations is also required to ensure shared investment in infrastructure, rewards, and educational campaigns. These partnerships reduce financial burdens placed on communities and provide technical support to maintain system functionality. Flexible incentive models also ensure sustainability by enabling the incentive scheme to be adapted to fit local needs—such as financial incentives, social recognition, vouchers, or access to community services—ensuring relevance and attractiveness to diverse user groups. Circonomy is also built to operate at various scales, from small communities to large urban areas, and it can gradually integrate additional technologies and features as resources grow.

Local resource utilization (materials and services for system maintenance, hardware replacement, and community events) is also important as a sustainability strategy. This not only reduces costs but also stimulates local economies.

Continuous community education is crucial to sustaining participation. Regular workshops, digital campaigns, and school programs promote an understanding of the circular economy and reinforce the value of waste sorting and recycling. Last but not least, aligning Circonomy with national and local regulations is crucial to ensuring legal support and access to public funding programs that can assist in long-term sustainability.

#### Alignment With Global Initiatives

Circonomy aligns directly with several global sustainability frameworks, notably the United Nations Sustainable Development Goals (SDGs), providing concrete contributions toward achieving global sustainability targets: (1) SDG 11 (Sustainable Cities and Communities): Circonomy facilitates sustainable urban environments by optimizing local waste management infrastructure through smart, community-centered approaches. Improving waste segregation accuracy and operational efficiency contributes to building sustainable and resilient urban communities. (2) SDG 12 (Responsible Consumption and Production): By integrating IoT-driven real-time monitoring, gamification-based incentives, and semantic web data management, Circonomy promotes responsible consumption habits and waste reduction among citizens. Its structured incentive framework directly encourages the adoption of recycling and reusing behaviors, aligning individual actions with broader circular economy principles. (3) SDG 13 (Combat Climate and Its Effect): Circonomy promotes 3R principles, preventing organic waste from being mixed with recyclables. This reduces emissions from landfills, a significant contributor to climate change.

#### Comparison With Other Global Waste Management Systems

After having several comprehensive discussions, we determined that it is important to compare and analyze how Circonomy can be applied not only as a potential solution for the local and national context but also for the global context. We conducted a structured comparison of traditional waste banks (Indonesia), Circonomy (Indonesia), Reciclos (Spain) [8], and Germany’s Deposit Refund Scheme [7] in terms of their mechanisms, incentives, technology used, gamification, impact, scalability, challenges, alignment with global waste management, and regulatory framework. Table 4 shows how the Circonomy features compare to these other waste management systems.

**Table 4.** Comparison table of Circonomy with the traditional waste bank in Indonesia, Reciclos (Spain), and Deposit Refund Scheme (Germany).

Feature	Traditional waste banks (Indonesia)	Circonomy (Indonesia)	Reciclos (Spain) [8]	Deposit Refund Scheme (Germany) [7]
Mechanism	Users bring recyclable materials to a Waste Bank, which weighs and records their contributions manually.	Uses IoT-enabled smart bins to monitor waste disposal; integrates gamification with points and leaderboards.	Uses a mobile app and QR codes to track recycling activity and reward participation with digital tokens.	A deposit fee is added to beverage purchases, refunded upon returning the container.
Incentives	Users receive money in exchange for recyclable materials, but prices fluctuate based on market demand.	Points-based system for waste disposal, with rewards for users and businesses.	Lottery-based system where users can exchange digital tokens for prizes or public benefits.	Financial incentive (deposit refund) for each returned container.
Technology used	Mostly manual processes, with some using basic digital weighing systems for record-keeping.	IoT-enabled smart bins, mobile app, and semantic web integration for waste tracking.	Mobile web app, blockchain-based smart contracts, and digital token system.	Simple barcode scanning system at return points, integrated with retailers.
Gamification	No structured gamification, but some waste banks provide community recognition programs.	Yes, with leaderboards, rewards for individuals and businesses, and interactive challenges.	Yes, lottery-based incentive program with digital tokens.	No direct gamification, but monetary rewards encourage participation.
Impact on recycling behavior	Encourages community participation in recycling but relies on voluntary participation and fluctuating material prices.	Encourages responsible waste disposal through automation and competition.	Increases recycling rates through incentive-based engagement.	Ensures high return rates for beverage containers, reducing littering.
Scalability	Limited scalability; depends on local community participation and funding.	Designed for urban environments with IoT infrastructure but faces challenges in areas with limited internet access.	Easily scalable due to mobile-based participation; requires local government support.	Highly scalable, already implemented nationwide with success.
Challenges	Dependent on fluctuating recyclable material prices; often lacks proper funding and long-term sustainability.	Infrastructure dependency (IoT and internet connectivity); sensor inaccuracies; needs more extensive gamification features.	User engagement depends on prize incentives; blockchain-based reward system requires public trust.	Administrative and logistical costs; effectiveness relies on high participation and proper waste sorting.
Alignment with circular economy	Moderate, as it promotes recycling but lacks systemic optimization and technology integration.	Strong, integrates smart technology for waste monitoring and promotes long-term behavior change.	Strong, supports a digitalized circular economy by incentivizing proper waste sorting and recycling.	Strong, creates a closed-loop system for beverage containers, ensuring material reuse.
Regulatory framework	Supported by the Indonesian government regulation about waste banks.	Supported by the Indonesian government regulation about waste banks but there is a lack of financial support from government and private sector partnerships.	Supported by the Spanish government and private sector partnerships (Ecoembes).	Mandated by German law; highly structured under Extended Producer Responsibility.

The table comparison illustrates that Circonomy bridges the gap between traditional and technology-driven waste management by integrating IoT, gamification, and the semantic web. Unlike traditional waste banks in Indonesia, which rely on manual tracking, Circonomy automates waste monitoring using IoT sensors, ensuring real-time tracking of bin capacity, waste weight, and user participation. Compared to Reciclos in Spain and Germany’s Deposit Refund Scheme, which focus mainly on plastic and beverage containers, Circonomy supports multimaterial waste sorting (plastic, organic, paper, metal, and glass) and assigns dynamic reward values to encourage responsible disposal. Gamification features such as leaderboards and digital incentives enable Circonomy to engage communities more effectively than existing systems, making waste management interactive and rewarding. Circonomy provides scalability and accessibility by using low-cost IoT components, making it more adaptable to regions with limited infrastructure than Germany’s Deposit Refund Scheme, which requires expensive reverse vending machines.

### Conclusion and Future Works

Circonomy has been implemented and evaluated from technical and user experience aspects. As an initial prototype, Circonomy has at least an 80% accuracy rate to ensure the model works reliably under real-world conditions while allowing room for incremental improvements and further interdisciplinary collaboration. Circonomy has potential as a solution encouraging circular waste management using technological innovation and socioenvironmental intervention, as it is aligned with research in [30]. Circonomy is grounded in 3 primary fields of study: computer science and engineering, social sciences, and environmental management. It serves as a potential circular waste solution not only in local and national contexts but also in a global context to address sustainable waste management challenges by moving toward a robust circular economy.

Future development of the Circonomy model will prioritize enhancing the accuracy and reliability of sensor-based occupancy detection through improved sensor placement, multiple sensor integration, and exploring alternative technologies for regions with limited IT resources. Additional features will be introduced to the gamification component, including interactive challenges and quizzes to educate users, strengthen community involvement, and reinforce long-term sustainable waste management behaviors. Further integration of semantic web technologies will optimize data management and facilitate better decision-making by effectively linking waste-related data, user actions, and recycling processes across digital platforms.

## Abbreviations

3R: reduce, reuse, recycle
API: application programming interface
DSRM: Design Science Research Methodology
IoT: Internet of Things
SDG: Sustainable Development Goal
